# Pharmacovigilance Agreements: Negotiating Safety Data Exchange Timelines: To Agree to Disagree? That is the Question

**DOI:** 10.1007/s43441-023-00543-z

**Published:** 2023-06-21

**Authors:** Wendy Manko Singer, Elizabeth MacEntee Pileggi, Yvonne Gibble, David John Lewis

**Affiliations:** 1 Individual Case Medical Review, Global Clinical Safety & Pharmacovigilance, Merck, Sharp & Dohme, LLC, Rahway, NJ USA; 2 Global Case and Safety Data Management, Global Medical Safety, Janssen, The Pharmaceutical Companies of Johnson & Johnson, Horsham, PA USA; 3 Pharmacovigilance Partner Strategy and Management, Global Clinical Safety & Pharmacovigilance, Merck Sharp & Dohme LLC, Rahway, NJ USA; 4 Global Drug Development, Novartis Pharma GmbH, Wehr, Germany; 5 School of Life and Medical Sciences, University of Hertfordshire, Hatfield, England

**Keywords:** Pharmacovigilance, Regulations, Contracts, Data management, Mandatory reporting, Safety data exchange agreements

## Abstract

Pharmaceutical companies often enter into contractual arrangements with other companies to advance the development or expand patient access of licensed medicines. These partnerships include specific agreements detailing the exchange of safety-related data between the companies. Such agreements are used to fulfil regulatory reporting obligations, thereby ensuring timely awareness of potential safety considerations and formal maintenance of clinical trial applications and marketing authorisations. The authors conducted potentially the first benchmarking survey of contracts covering safety data exchange within the pharmaceutical industry. Data were analysed to establish the most common types of safety data exchanged, and the associated data exchange timelines. These data may provide an opportunity for companies to assess how their own timelines compare with others, and to consider whether there are actions they may take that could potentially improve negotiation and procedural efficiency. Ninety percent of the recipients responded to the survey, providing information from 378 individual contracts which included data from clinical trials and from postmarketing sources. Results showed less variability in the safety data exchange timelines of clinical trial ICSRs compared to the timelines of postmarketing ICSRs; these results may reflect greater harmonisation of regulatory reporting requirements for clinical trials. The variability captured in the benchmarking data reflects the challenges that contribute to the complexity of safety data exchange agreements between partner companies. The goal of the survey was to serve as a basis for future research and pursuit of additional insights that foster transparency. The aim was also to encourage the consideration of alternative approaches to address some of the challenges that we identified. Use of technology could facilitate the process of recording, tracking, and monitoring of safety data exchange within a partnership, improve efficiency through real time monitoring, and provide further insights. A proactive approach to developing agreements is essential for improved patient access and to maintain patient safety.

## Introduction

There is an increasing trend for pharmaceutical companies to partner on common strategic priorities for licensed medicinal products, such as research and development initiatives and establishing a presence in global markets. According to industry leaders, “partnering and collaborating in a new way is the only means by which the industry and the healthcare sector as a whole can have a meaningful impact on the health of the nation and the world” [1].

The ways in which pharmaceutical companies work together vary depending on their goals. Some common examples include marketing the same drug in different regions or codevelopment and joint ventures in clinical trial collaborations. In 2020, there were at least 108 pharmaceutical and life sciences transactions announced, 256 transactions announced in 2021, and 46 transactions announced during the first 6 months of 2022 [2]. Indeed, the changing landscape, unprecedented medical needs, and complexity of therapies in emerging areas, such as cancer and COVID-19, have resulted in partnerships between the pharmaceutical industry, government agencies, and/or other institutions to develop treatments. Additionally, business partnerships continue to be a large value driver for biopharmaceutical companies to fill or augment pipelines, increase revenue, acquire gene and cell technology, and ultimately to address unmet patient needs.

While collaboration opportunities for pharmaceutical companies have increased, the literature on this innovative way of working is limited, and regulations that define how to share information in these relationships lack clarity and harmonization, allowing for differing interpretations. What health authorities do make clear is that, regardless of each company’s responsibilities in an agreement, companies must comply with the regulatory reporting requirements for safety data (e.g., individual case safety reports [ICSRs], aggregate safety data reports). As such, health authorities can significantly impact the way pharmaceutical companies partner together, underscoring the importance of detailed procedural documentation for safety data exchange.

For example, the European Medicines Agency guideline on good pharmacovigilance practices Module VI specifies that, “Where the marketing authorisation holder has set up contractual arrangements with a person or an organisation, explicit procedures and detailed agreements should exist between the marketing authorisation holder and the person/organisation to ensure that the marketing authorisation holder can comply with the submission of valid ICSRs within the appropriate time frames. These procedures should in particular specify the processes for the exchange of safety information, including the timelines and responsibilities for the regulatory submission of valid ICSRs. They should be organised in order to avoid the submission of duplicate ICSRs to the competent authorities” [4].

When a pharmaceutical company enters a contractual arrangement with another company, a separate Pharmacovigilance Agreement (PVA)/Safety Data Exchange Agreement is established to specify the details of safety-related data that will be exchanged between the companies. Specifically, these agreements are used to fulfil regulatory reporting obligations for medicinal products in the countries where one of the companies holds a clinical trial application or marketing license.

While the nuanced details of a PVA will vary with the type of agreement involved (e.g., in-licensing, out-licensing, codevelopment, comarketing, distribution, copromotion), a PVA must include details of the collaboration, roles, and responsibilities, and committed obligations between the partners on critical safety-related topics, such as timelines and safety data exchange format. However, there can be large variability in how companies process safety data. Notable factors that can contribute to the complexity of PVAs and priorities of the partnering companies include company size, product type and development phase, the number of partner companies, and whether certain or all aspects of safety data processing are conducted in-house or outsourced.

Importantly, the timelines for safety data exchange between companies must be defined in an agreement for all parties involved to support compliance with regulatory reporting requirements. Differences in the procedures, processes, commitments, and constraints of the contractual partners can lead to difficulties in negotiating these timelines. Indeed, negotiating data exchange timelines, as well as what types of safety data to exchange, often take a significant amount of time and effort for partnering companies to align.

When, where, and how different companies process safety data remain unclear since this internal company information is not typically shared. Consequently, this lack of transparency may be one reason why companies initially have differing perspectives on the appropriate data exchange timeframes when entering new partnerships. Companies can also have unique operating procedures and internal timelines in place for processing safety data based on their internal business structure. Receiving safety data outside the company’s operational window can negatively impact their ability to meet regulatory reporting deadlines and potentially delay identification of potential safety concerns. Thus, while often difficult, the negotiation and agreement of safety data exchange timelines between partners is pivotal to maintaining regulatory compliance and patient safety.

To address the lack of industry transparency and limited literature on safety data sharing and reporting in pharmaceutical partnerships, members of the TransCelerate Pharmacovigilance Agreement Optimization initiative [5] conducted a PVA benchmarking survey focusing on the common types of safety data companies agreed to exchange and the agreed upon data exchange timelines. The goal was to provide insight into timelines used within the industry and make the survey data publicly available. Publishing the survey data was not intended to provide recommendations on any safety data exchange timelines. The survey data may, however, provide an opportunity for companies to assess how their own timelines compare and consider whether there are actions they can take in their own processes that could potentially improve negotiation and/or procedural efficiency.

## Methods

### Data Collection

A benchmarking survey was distributed to 20 TransCelerate Member Companies (MC) inviting them to voluntarily provide data from up to 30 PVAs regarding common safety data exchange timelines, including ICSRs, between companies that enter a PVA. The survey data were anonymized and aggregated to maintain confidentiality of survey participants. Only the portions of the survey related to ICSR exchange for clinical trials and postmarketing sources (e.g., spontaneous and solicited/non-interventional studies) are described here. Survey questions used for provision of the data in this paper are provided in the Supplementary Information.

The metric gathered on the ICSR exchange timeline was the number of calendar/business days for the exchange of ICSR data between partners; this metric was provided for the directional data exchange from MC to partner (P) and from P to MC. Survey questions were designed to enable MCs to indicate case types. For clinical trial ICSRs, directional exchange timelines were reported for the following case types: related fatal/life-threatening serious adverse events (SAEs) and related non-fatal/life-threatening SAEs. For postmarketing ICSRs, directional exchange timelines were reported for the following case types, irrespective of relatedness: serious spontaneous or solicited adverse events and non-serious spontaneous or solicited adverse events.

### Data Analyses

The results presented in this paper are summary measures limited to descriptive statistics for “company-level” analyses and “PVA-level” analyses of the ICSR exchange timeline data; no formal statistical hypothesis testing was performed. “Company-level” analyses correspond to the data obtained from the benchmarking survey based on the totality of the number of MCs that responded to each question. “PVA-level” analyses correspond to the data obtained from the benchmarking survey based on the totality of the number of PVAs the MCs used to respond to each question. To mitigate the potential of data bias, each MC was asked to provide data from a maximum of 30 company PVAs; this upper cap was established to prevent the data from being disproportionally driven by any one MC. Conversely, when fewer than five MCs provided information for a particular ICSR exchange day, the data are not reported.

“Company-level” clinical trial ICSR data and postmarketing ICSR data were reviewed. The most common directional exchange days across MCs were identified. For the clinical trial ICSR data, each MC was counted once if at least one PVA required ICSR exchange on that day. The frequency of MCs that required ICSR exchange on a particular day is reported only if at least five MCs are represented; thus, exchange days that have no MC counts indicate that less than five MCs or no MCs required exchange on that day. The cut point of five was chosen based on subject matter expertise and the balance of the data. Summary “company-level” data review centered on the two most common directional exchange days; this includes the number of PVAs in the summary, which indicates the total number of instances where a MC has at least one of their PVAs requiring data exchange on any day (including less common exchange days that were not reported).

For the postmarketing ICSR data, the “company-level” data indicated that some MCs may differentiate postmarketing exchange days based on case type (i.e., spontaneous or solicited). For each exchange day and potential case type differentiation, each MC was counted once if at least one PVA required exchange on that day and that specific case type. The frequency of MCs that required ICSR exchange on a particular day is reported only if at least five MCs required exchange in at least one of their PVAs for that day; thus, exchange days that have no MC counts indicate that less than five MCs or no MCs required exchange on that day.

“PVA-level” summary data are presented only for the clinical trial ICSRs. For the “PVA-level” data the most common directional exchange days across all individual PVAs were identified, regardless of which MC submitted the PVA. The total number of PVAs counted for MCs that provided an exchange day for at least one of their PVAs. The data were evaluated so that no one MC accounted for more than about 25% of the total and are reported only when a minimum of five MCs contributed to the summary. The percentage of most common exchange days is out of the total number of PVAs.

## Results

Ninety percent of the surveyed MCs (18/20) responded to the benchmarking survey, providing information from 378 individual PVAs. Of the 378 PVAs, 267 provided clinical trial timelines and 326 provided postmarketing timelines (including solicited and spontaneous). Data from clinical trial ICSRs with related fatal/life-threatening SAEs and related non-fatal/life-threatening SAEs as well as from postmarketing ICSRs with serious solicited or spontaneous adverse events and non-serious solicited or spontaneous adverse events are described below.

### Clinical Trial ICSRs with Related Fatal/Life-Threatening SAEs

**Fig. 1:**
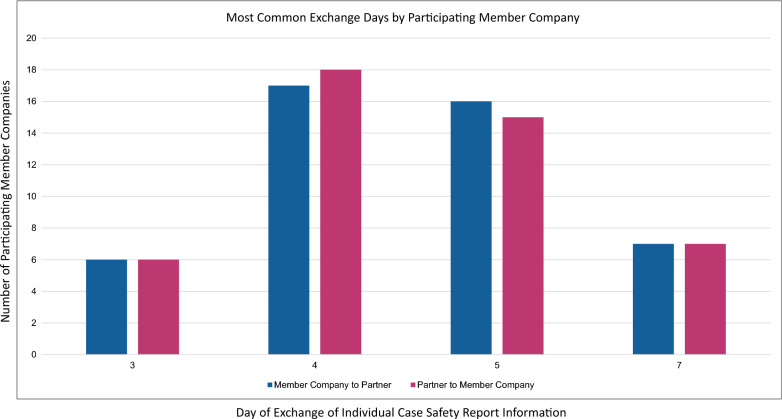
Company-level analysis: most common data exchange days for clinical trial ICSRs with related fatal/life-threatening SAEs reported by member companies (MCs). The data represent the exchange day (x-axis) per total number of MCs that required data exchange on that day (y-axis). Directional data exchange from MC to partner is depicted in blue and directional data exchange from partner to MC is depicted in pink

The “company-level” data for clinical trial ICSRs with related fatal/life-threatening SAEs are summarized in Fig. 1 and Table 1. For the directional data exchange from MC to P, the most common exchange days were Days 3, 4, 5, and 7 (Fig. 1) and the approximate exchange day probable range is from 1.6 lower confidence limit (LCL) to 10.1 upper confidence limit (UCL) (Table 1). Forty-seven percent of the total number of PVAs were on or before Day 4 and 53% of the total number of PVAs were on or after Day 5 (Table 1). For the directional data exchange from P to MC, the most common exchange days were Days 3, 4, 5, and 7 (Fig. 1) and the approximate exchange day probable range is from 1.1 (LCL) to 13.4 (UCL) (Table 1). Fifty percent of the total number of PVAs were on or before Day 4 and 50% of the total number of PVAs were on or after Day 5 (Table 1).

**Table 1:** Company-level Analysis: Summary of the Data Exchange Days for Clinical Trial ICSRs with Related Fatal/Life-threatening SAEs Reported by Member Companies

	Member company to partner		Partner to member company	
	On or before Day 4	On or after Day 5	On or before Day 4	On or after Day 5
Number of participating member companies	18		18	
Number of PVAs in this summary	57		62	
Percentage of PVAs	47%	53%	50%	50%
Median exchange day	4.0	5.0	4.0	6.0
Mean exchange day	3.4	6.4	3.2	7.1
Standard deviation	0.9	1.9	1.1	3.3
Lower confidence limit	1.6		1.1	
Upper confidence limit		10.1		13.4

The “PVA-level” data for clinical trial ICSRs with related fatal/life-threatening SAEs is summarized in Table 2. For the directional data exchange from MC to P, there were 246 individual PVA responses. The most common exchange days were Days 4 and 5, with 79% of the total number of PVAs on Day 4 or 5 (the remainder of PVAs had exchange days either before Day 4 or after Day 5). For the directional data exchange from P to MC, there were 245 individual PVA responses. The most common exchange days were Days 4 and 5, with 79% of the total number of PVAs on Day 4 or 5 (the remainder of PVAs had exchange days either before Day 4 or after Day 5).

**Table 2:** PVA-level analysis: most common data exchange days for clinical trial ICSRs with related fatal/life-threatening SAEs reported by member companies

	Member company to partner	Partner to member company
Total number of PVAs	246	245
Most common exchange days (ascending order)	4, 5	4, 5
Percent of PVAs with exchange on most common exchange days	79%	79%

### Clinical Trial ICSRs with Related Non-fatal/Life-Threatening SAEs

**Fig. 2:**
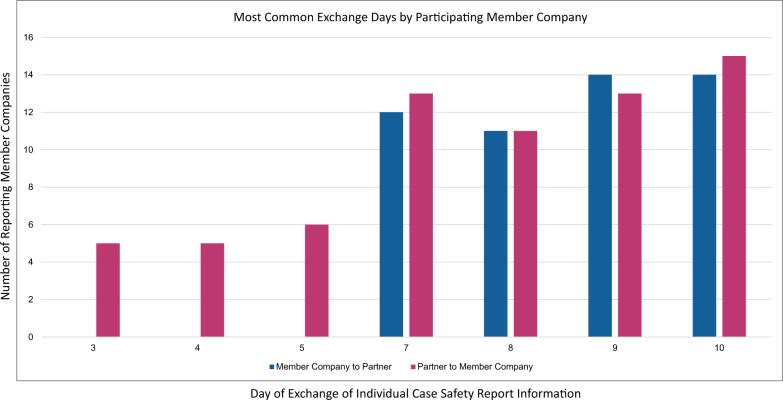
Company-level analysis: most common data exchange days for clinical trial ICSRs with related non-fatal/life-threatening SAEs reported by member companies (MC). The data represent the exchange day (x-axis) per total number of MCs that required data exchange on that day (y-axis). Directional data exchange from MC to partner is depicted in blue and directional data exchange from partner to MC is depicted in pink

The “company-level” data for clinical trial ICSRs with related non-fatal/life-threatening SAEs are summarized in Fig. 2 and Table 3. For the directional data exchange from MC to P, the most common exchange days were Days 7, 8, 9, and 10 (Fig. 2) and the approximate exchange day probable range is from 2.4 (LCL) to 20.2 (UCL) (Table 3). Sixty-eight percent of the total number of PVAs were on or before Day 9 and 32% of the total number of PVAs were on or after Day 10 (Table 3). For the directional data exchange from P to MC, the most common exchange days were Days 3, 4, 5, 7, 8, 9, and 10 (Fig. 2) and the approximate exchange day probable range is from 2.0 (LCL) to 16.5 (UCL) (Table 3). Approximately 72% of the total number of PVAs were on or before Day 9 and 28% of the total number of PVAs were on or after Day 10 (Table 3).

**Table 3:** Company-level Analysis: summary of the data exchange days for clinical trial ICSRs with related non-fatal/life-threatening SAEs reported by member companies

	Member company to partner		Partner to member company	
	On or before Day 9	On or after Day 10	On or before Day 9	On or after Day 10
Number of participating member companies	18		18	
Number of PVAs in this summary	75		78	
Percentage of PVAs	68%	32%	72%	28%
Median exchange day	7.0	10.0	7.0	10.0
Mean exchange day	6.8	12.0	6.5	11.3
Standard deviation	2.3	4.2	2.3	2.7
Lower confidence limit	2.4		2.0	
Upper confidence limit		20.2		16.5

The “PVA-level” data for clinical trial ICSRs with related non-fatal/life-threatening SAEs is summarized in Table 4. For the directional data exchange from MC to P, there were 248 individual PVA responses. The most common exchange days were Days 8, 9, and 10, with 72% of the total number of PVAs on Days 8, 9, or 10 (the remainder of PVAs had exchange days on days other than Days 8, 9, or 10). For the directional data exchange from P to MC, there were 251 individual PVA responses. The most common exchange days were Days 8, 9, and 10, with 69% of the total number of PVAs on Days 8, 9, or 10 (the remainder of PVAs had exchange days on days other than Days 8, 9, or 10).

**Table 4:** PVA-level analysis: most common data exchange days for clinical trial ICSRs with related non-fatal/life-threatening SAEs reported by member companies

	Member company to partner	Partner to member company
Total number of PVAs	248	251
Most common exchange days (ascending order)	8, 9, 10	8, 9, 10
Percent of PVAs with exchange on most common exchange days	72%	69%

### Postmarketing ICSRs with Serious Solicited or Spontaneous Adverse Events

**Fig. 3:**
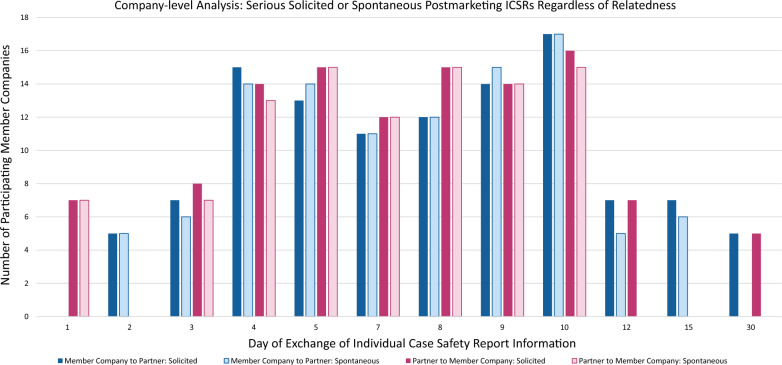
Company-level analysis: most common data exchange days for postmarketing ICSRs with serious solicited or spontaneous adverse events (Regardless of Relatedness) reported by member companies

The “company-level” data for postmarketing ICSRs with serious solicited or spontaneous adverse events is summarized in Fig. 3. The “company-level” summary is displayed regardless of the relatedness and therefore includes the combination of both related and not-related timelines.

For the directional exchange of solicited data from MC to P, the most common exchange days were Days 2, 3, 4, 5, 7, 8, 9, 10, 12, 15, and 30. For the directional exchange of spontaneous data from MC to P, the most common exchange days were Days 2, 3, 4, 5, 7, 8, 9, 10, 12, and 15.

For the directional exchange of solicited data from P to MC, the most common exchange days were Days 1, 3, 4, 5, 7, 8, 9, 10, 12, and 30. For the directional exchange of spontaneous data from P to MC, the most common exchange days were Days 1, 3, 4, 5, 7, 8, 9, and 10.

### Postmarketing ICSRs with Non-serious Solicited or Spontaneous Adverse Events

**Fig. 4:**
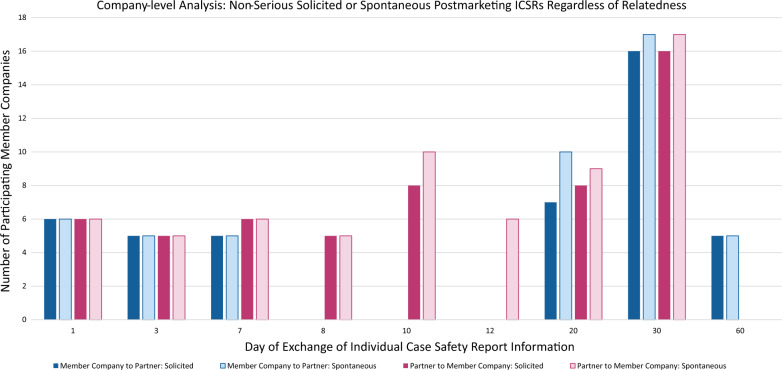
Company-level analysis: most common data exchange days for postmarketing ICSRs with Non-serious solicited or spontaneous adverse events (Regardless of Relatedness) reported by member companies

The “company-level” data for postmarketing ICSRs with non-serious solicited or spontaneous adverse events is summarized in Fig. 4. The “company-level” summary is displayed regardless of the relatedness and therefore includes the combination of both related and not-related timelines.

For the directional exchange of solicited data from MC to P, the most common exchange days were Days 1, 3, 7, 20, 30, and 60. For the directional exchange of spontaneous data from MC to P, the most common exchange days were Days 1, 3, 7, 20, 30, and 60.

For the directional exchange of solicited data from P to MC, the most common exchange days were Days 1, 3, 7, 8, 10, 20, and 30. For the directional exchange of spontaneous data from P to MC, the most common exchange days were Days 1, 3, 7, 8, 10, 12, 20, and 30.

## Discussion

To our knowledge, this is the first benchmarking survey on safety data exchange timelines in the pharmaceutical industry. Overall, the data showed less variability in the safety data exchange timelines of clinical trial ICSRs compared to the timelines of postmarketing ICSRs; these results may reflect the regulatory agencies’ reporting requirements for clinical trial ICSRs. The variability captured in the benchmarking data may also reflect the notable challenges that can contribute to the complexity of PVAs and negotiating safety data exchange timelines between partnering companies.

### Clinical Trial Partnership Challenges

**Fig. 5:**
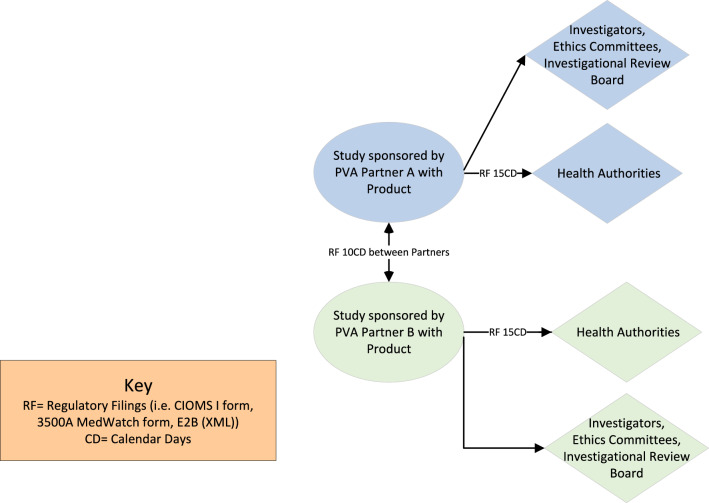
Example of a safety data exchange timeline between two sponsors in a clinical trial partnership wherein both sponsors share similar data processing and reporting responsibilities

Clinical trial sponsors are responsible for all regulatory reporting under the investigational product application. Therefore, partnerships involving two clinical trial sponsors can be streamlined and often easier to negotiate ICSR exchange timeframes as there are clear and concise regulations governing clinical trial sponsors, particularly when both partners share the safety data processing duties (Fig. 5). However, if one partner assumes sponsor responsibilities for ICSR processing, the delegating sponsor is not relieved of their regulatory obligations. As such, this latter scenario could add complexity to the data exchange negotiation process because the delegating sponsor must continue to ensure their regulatory obligations are met.

### Postmarketing Partnership Challenges

**Fig. 6:**
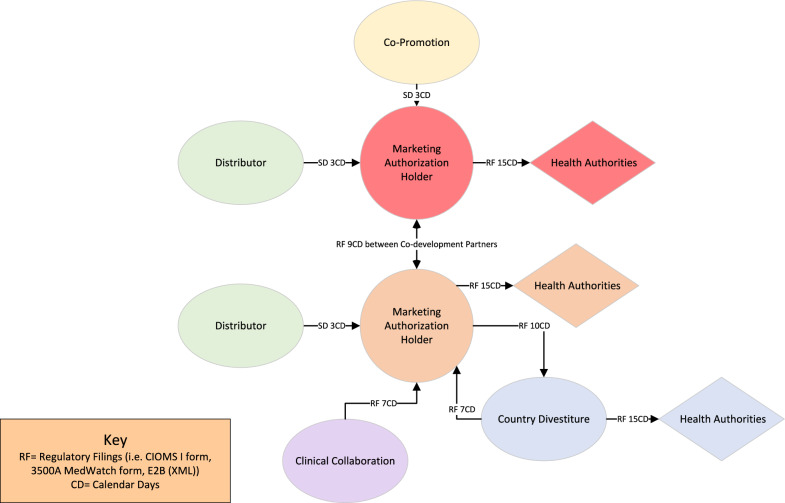
Example of safety data exchange timelines between multiple sponsors in a postmarketing partnership involving approved products wherein sponsors assume different data processing and reporting responsibilities

**Fig. 7:**
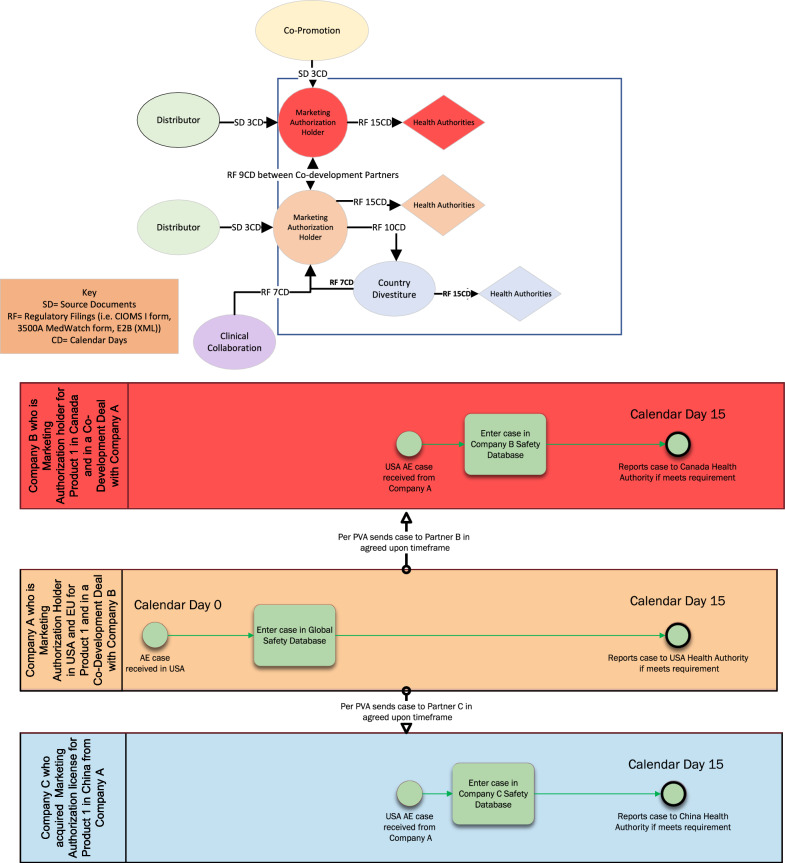
Detailed hypothetical example of safety data exchange timelines between multiple sponsors in a postmarketing partnership involving approved products wherein sponsors assume different data processing and reporting responsibilities

In postmarketing relationships, there are more opportunities for variation in the structure of a partnership within the approved product licensure (Figs. 6, 7). PVAs may cover commercially available products that allow for product use within the approved status or may include partnerships focused on making commercial products available to patients in clinical trials. Similarly, the role of the partner in the PVA can vary from a limited role of product distributor to holding the marketing authorization in a specific country/region.

The marketing authorization holder (MAH) of a commercially approved product can enter different relationships where a partner is performing the responsibility for all safety data processing activities or sharing responsibilities that could result in partners having the same or different regulatory obligations. For example, in the instance where one partner does not have direct regulatory agency reporting responsibility (e.g., distributor) but is limited to providing the MAH partner with data, this often translates to shorter timelines for the former partner to allow the MAH partner to fulfill their reporting responsibilities. There are other relationships wherein both partners have an MAH, in either the same territory (under different brand names) or different territories (that may or may not have the same brand name). This scenario could result in complicated timeline negotiations because of the need for both MAH partners to receive safety data within specified timelines to meet their surveillance and reporting obligations.

### Operational Tolerance Challenges

Regardless of the relationship structure, partnering companies may prefer different safety data exchange timelines to support their individual business needs. These needs may be influenced by differing organizational processes or operational tolerance for managing negotiated timelines that may result in process exceptions. For example, a partner with an organizational model that requires forwarding of data to a different group within their organization or to an external strategic partner may require data receipt in shorter timelines to allow for this additional activity. Adding non-standard process activities to address obligations within a partnership can add complexity and challenges with determining acceptable data exchange timelines for both partners. For instance, when an MAH partner delegates an obligation to a partner (e.g., submit ICSRs to a regulatory agency in the partner’s territory), this may require an additional activity to be incorporated into their organizational processes (e.g., approval of an ICSR by the MAH prior to submission by their partner on their behalf).

### Interpretation of Regulations and Guidance Challenges

An additional challenge in PVA negotiation may arise when the partners have different interpretations of regulatory agency requirements and philosophies regarding MAH assessment and reporting obligations. This can be a challenge particularly when one partner receives data from a partner territory and must determine if they are willing to accept the codification and associated assessment of data provided by the partner. In this case, shorter data receipt timeframes may be required to allow for their internal reassessment of the data. One of the most compelling examples suggestive of divergent interpretation of regulatory requirements was the non-serious postmarketing data summary analyses. There was a widespread range [See Fig. 4 Postmarketing ICSRs with Non-serious Solicited or Spontaneous Adverse Events] to suggest a lack of harmonization on the interpretation of health authority requirements. According to Article 107(3) and 107a(4) of Directive 2001/83/EC, “serious valid ICSRs shall be submitted by the competent authority in a Member State or by the marketing authorisation holder within 15 days from the date of receipt of the reports; non-serious valid ICSRs shall be submitted by the competent authority in a Member State or by the marketing authorisation holder within 90 days from the date of receipt of the reports” [5]. In this example, divergent interpretations of what constitutes a serious ICSR and non-serious ICSR and the required timelines based upon the assessment and interpretation of each partner, potentially impacts the timelines for exchanging of ICSRs. This regulation citation and the interpretation across the negotiating parties are an example of drivers that present challenges and add complexity when negotiating PVAs.

### Multiple Partners and Product Type Challenges

Innovative collaborations can involve multiple partnering companies. However, having more than two partners can increase the complexity of the clinical trial and relationship management as defined in the PVA.

The nature of the product and the relationship may bring additional considerations to timelines that must be managed between partners. A product with potential high volume data exchange (such as blockbuster products) or products with high exposure (such as large vaccination programs during a pandemic) could introduce unprecedented operational efficiency challenges. These challenges can impact the ability to exchange data in shorter timelines and may require innovative solutions, such as leveraging automation.

With recent developments in global healthcare needs, such as Ebola and the COVID-19 pandemic, there is a sense of urgency to move with improved speed and efficiency within the pharmaceutical industry. Working collaboratively with multiple partners is one approach to address this need but it can also impact the complexity of negotiating PVA timelines.

## Conclusion

The TransCelerate Pharmacovigilance Agreement Optimization initiative [4] conducted this PVA benchmarking survey to address the lack of published information about the contents of PVAs (e.g., ICSR exchange timelines). While the survey data provide some insights on the data exchange timelines used within the industry, data collection limitations should be noted. The survey was limited to ICSR exchange timelines for clinical trials and postmarketing sources; it did not collect information about which territories were covered, partnership details, the type and scope of the products involved, details about specific circumstances that may apply (e.g., actions included in response to regulatory findings), or involvement of tertiary parties and their effects on timelines.

Additional data limitations include the potential for under- or over-representation of a single day of exchange in the “company-level” assessment. For example, a single company may have contributed to most of the data on a given exchange day. To minimize potential over-representation, the data for that point represents the number of companies exchanging on that day regardless of the number of PVAs from the represented companies. Similarly, it is possible that all responding MCs on a given exchange day may have only one PVA that exchanged data on that day, but the majority of their PVAs could have been submitted on a different day. The “company-level” data bar charts therefore demonstrate the tolerance of a responding MC to exchange on a particular day, which may be based on a specific set of circumstances that are not transparent based on the data provided. Moreover, because the data was provided by responding MCs, the data may be limited by the sample size and characteristics of the participating companies. Timelines for data exchange could have also varied based upon partner relationship nuances. Limitations of the “PVA-level” data include those for the “company-level” data, but also include consideration that summary measures could be influenced by one or a small number of companies.

Finally, a limitation that applies to both the “company-level” and PVA-level" data is the possibility for differing interpretations of day zero, or the clock start date (CSD) for purposes of regulatory agency reporting, among different regulations and territories. Our benchmarking survey did not include questions regarding a company’s interpretation or definition of the CSD, nor did it consider the different possible interpretations of regulations by a responding MC across the globe. PVAs involving partners who have regulatory agency reporting in countries with different requirements for how the CSD is defined may find this is a key factor in negotiating data exchange timeframes. Japan, for example, allows for a new CSD to be assigned at the time the Japanese partner company receives the ICSR and not when the first company obtained it. Conversely, other countries continue to use the CSD from the first partner company that received the information. Therefore, in this example, the countries with the most stringent timelines for submissions (i.e., earliest CSD received by any partner) may tend to drive the exchange days negotiated in the PVAs. Regardless of which countries regulate the CSD, data exchange timelines in PVAs will generally aim to meet the requirements of the most stringent regulations that help drive public health across the globe. PVAs that cover products authorized in multiple territories require negotiation of all details pertaining to timelines, as there may be a lack of regulatory harmonization; this becomes especially complicated in relationships with more than one partner.

The goal of the PVA benchmark information on ICSR exchange timelines is to serve as a basis for future research and pursuit of additional insights that foster transparency, and potentially encourage consideration of alternative approaches to address some of the PVA challenges that we have outlined. ICSR automation (or a similar technology) is one possible approach that could facilitate the process of recording, tracking, and monitoring of ICSR exchange within a partnership, improve efficiency through real time monitoring, and provide further insights. One potential adoption of advanced technology would be application of block chain, which is a distributed ledger technology that would enable ICSR traceability across both partners and provide transparency of data exchange. The implementation of blockchain is complex and requires financial investment from both parties. Alternative to exchanging data between parties could be a globally distributed sharing platform where the data is processed by the originating party and stored in a single repository with controlled access to the data limited to the contractual partners [6].

The potential insights gained from research in PVA negotiation and safety data exchange support exploring proactive approaches for safety data exchange in an environment with increasing regulatory complexity, novel product development, and partnerships. This proactive approach is critical for improved patient access and safety.

## Data Availability

The surveys conducted in this study are provided as supplemental materials. If necessary please include in the article where TIRS thinks it’s appropriate.
